# Editorial: Guidance of search by long-term and working memory

**DOI:** 10.3389/fcogn.2024.1495416

**Published:** 2024-10-02

**Authors:** Stefan Pollmann, Thomas Geyer, Jun Kawahara

**Affiliations:** ^1^Department of Experimental Psychology, Institute of Psychology, Faculty of Natural Sciences, Otto von Guericke University Magdeburg, Magdeburg, Germany; ^2^Department of Psychology, Ludwig Maximilian University of Munich, Munich, Bavaria, Germany; ^3^Department of Psychology, Hokkaido University, Sapporo, Hokkaido, Japan

**Keywords:** visual, search, memory, attention, learning

Visual search is often guided by memory. This is the topic of the present Research Topic. It contains a Research Topic of papers that investigate diverse issues in this field with behavioral measures. One frequently used paradigm to investigate memory-guided search is the contextual cueing paradigm, in which the repeated presentation of target-distractor configurations leads to incidental learning and subsequently faster search in repeated than novel displays. Among the papers of this Research Topic, four used variations of this paradigm.

Albert et al. looked into contextual cueing for two target locations in real-world scenes. They found search benefits for both targets, rejecting the hypothesis that contextual cueing of one target location comes at a cost for the other target location. After extensive training, even targets at new locations but within the same hemifield as the cued targets benefitted. Analyzing eye movements, they further found that contextual cueing led to reduced numbers of searching fixations, but not responding fixations, implying that the search benefit due to contextual cueing was due to attentional guidance rather than response-related processes.

von Mühlenen and Conci investigated how the presence of a task-irrelevant object influences the contextual cueing effect. In a series of experiments, they found that the addition of a green square to displays with a target “T” and distractor “Ls” could reduce the contextual cueing effect, particularly if the square was non-overlapping with the display items (not seen as a background item). Furthermore, the irrelevant objects interfered both with learning of repeated displays and with expression of learning, i.e., search guidance by learned displays.

Zinchenko et al. found that the effect of negative vs. neutral emotional stimuli presented immediately before search displays depended on their nature. While emotional faces had no significant effect on contextual cueing, emotional scenes increased contextual cueing. Moreover, individual differences were observed that were unlikely to be caused by valence or arousal variability. The study shows that the effects of emotional processes on contextual cueing are still a field that invites further research.

Zheng et al. investigated contributions of global configuration vs. individual spatial item position to contextual cueing. Replicating previous research, they found that search guidance based on either local or global spatial context, by combining distractor locations from two learned displays or rotating displays, led to search time facilitation. Reduced search times were accompanied by less fixations and more direct scan paths to the target. Moreover, fixation distribution maps of recombined or rotated displays were more similar to the original displays than random new displays. However, for rotated displays this was only true when the rotation angle was taken into account, implying a rotated fixation pattern. Overall, this shows an astonishingly flexible use of the oculomotor system for search in incompletely repeated displays.

Another study, by Barbosa et al., used the hybrid search paradigm, i.e., a combination of memory search and visual search. Importantly, in each trial, a new set of objects had to be memorized and subsequently, it had to be determined if a target item from the memory set was included in a visual search display. They replicated previous studies in that search times increased linearly with visual set size and logarithmically with memory set size. This pattern was preserved when a realistic context was added as background. Individual differences in inhibitory control and working memory capacity did little to explain hybrid search efficiency.

Finally, Büsel et al. used the contingent-capture paradigm, which presents a visual search task that is preceded by a color cue, in order to investigate whether the representation of visual information in a given search-guiding target template may also contain task-set specifications, i.e., information about the mapping of a given target (color) feature to a given (button press) response. They show that cue-target congruence effects scaled with variations of the task-sets, which suggests that response-related information is represented in these templates. The study thus provides an interesting novel perspective to the attentional capture literature.

In summary, these studies are all examples that visual search is guided by memory, but, as good research should, they also open new questions, inviting follow-up research. Of two targets are available, what determines which target becomes dominantly learned? How is this linked to memory representation, as Albert et al. ask? When does an irrelevant additional object interfere with contextual cueing? Does it depend on depth segregation, as von Mühlenen and Conci suggest? What causes the differential effect between emotional scenes and emotional faces on contextual cueing, observed by Zinchenko et al.? Is the rotated fixation pattern observed by Zheng et al. simply due to looking at a rotated display or is it due to search guidance by a mentally rotated memory pattern? Would a semantically meaningful background that interferes with the memorized objects change the findings by Barbosa et al.? These are among the questions raised by the papers in this Research Topic. May they lead to new studies that increase our insight into the mechanisms of memory-guided search.

